# H_2_S Protects against Cardiac Cell Hypertrophy through Regulation of Selenoproteins

**DOI:** 10.1155/2019/6494306

**Published:** 2019-09-10

**Authors:** Adam Greasley, Yanjie Zhang, Bo Wu, Yanxi Pei, Nelson Belzile, Guangdong Yang

**Affiliations:** ^1^Department of Chemistry and Biochemistry, Laurentian University, Sudbury, Canada; ^2^Cardiovascular and Metabolic Research Unit, Laurentian University, Sudbury, Canada; ^3^School of Life Science, Shanxi University, Taiyuan, China; ^4^Department of Pathophysiology, Harbin Medical University, Harbin, China

## Abstract

Cardiac hypertrophy is defined as the enlargement of the cardiac myocytes, leading to improper nourishment and oxygen supply due to the increased functional demand. This increased stress on the cardiac system commonly leads to myocardial infarction, contributing to 85% of all cardiac-related deaths. Cystathionine gamma-lyase- (CSE-) derived H_2_S is a novel gasotransmitter and plays a critical role in the preservation of cardiac functions. Selenocysteine lyase (SCLY) has been identified to produce H_2_Se, the selenium homologue of H_2_S. Deficiency of selenium is often found in Keshan disease, a congestive cardiomyopathy. The interaction of H_2_S and H_2_Se in cardiac cell hypertrophy has not been explored. In this study, cell viability was evaluated with a 3-(4,5-dimethylthiazol-2-yl)-2,5-diphenyltetrazolium bromide (MTT) assay. Oxidative stress and cell size were observed through immunostaining. The expression of genes was determined by real-time PCR and western blot. Here, we demonstrated that incubation of rat cardiac cells (H9C2) with H_2_O_2_ lead to increased oxidative stress and cell surface area, which were significantly attenuated by pretreatment of either H_2_S or H_2_Se. H_2_S incubation induced SCLY/H_2_Se signaling, which next caused higher expressions and activities of selenoproteins, including glutathione peroxidase and thioredoxin reductase. Furthermore, deficiency of CSE inhibited the expressions of SCLY and selenoprotein P in mouse heart tissues. We also found that both H_2_S and H_2_Se stimulated Nrf2-targeted downstream genes. These data suggests that H_2_S protects against cardiac hypertrophy through enhancement of a group of antioxidant proteins.

## 1. Introduction

Cardiovascular disease (CVD) is a leading cause of death world-wide contributing to approximately 31% of all deaths annually. More than 85% of all CVD-related deaths are contributed to or caused by heart attacks and strokes, both of which are typical end results of chronic pathologies, such as cardiac hypertrophy [1]. Cardiac hypertrophy is both a natural and responsive change where the myocardium undergoes overgrowth in response to external and internal stimuli, such as reactive oxygen species (ROS) or pressure overload [1, 2]. An increase in heart size is accompanied by a high demand of oxygen and nutrients to sustain function. In cases where the oxygen and nutrient demand is not met, myocardial ischemic conditions persist, which will result in cardiac cell death, tissue fibrosis, and subsequent cardiac infarcture [3]. Two fetal genes atrial natriuretic factor (ANF) and brain natriuretic peptide (BNP) have long been used as molecular markers for the diagnosis of pathological hypertrophy [3–5].

Hydrogen sulfide (H_2_S) is a highly diffusible molecule and classified as a novel gasotransmitter along with nitric oxide and carbon monoxide [6–9]. H_2_S can be produced endogenously in our cells through cystathionine gamma-lyase (CSE), cystathionine beta-synthetase (CBS), and/or 3-mercaptopyruvate sulfurtransferase (3-MST) [10, 11]. The concentration of H_2_S is not homogenous throughout different tissues; certain tissues have higher production rates such as the liver and vasculature, when compared to other tissues such as neuronal [10]. This difference in production affects the distribution of H_2_S-producing enzymes throughout the body; CSE has the greatest H_2_S-producing ability through the catalysis of L-cysteine (Cys) to H_2_S [8, 12]. H_2_S levels in the vasculature have been estimated to be somewhere from 10 to 100 *μ*M with initial techniques; however, more recently, there has been controversial evidence of levels in the low nanomolar range [10, 11]. In the vasculature, CSE is the dominant enzyme for H_2_S production, where CSE knockout mice show markedly decreased plasma H_2_S levels and cardiac dysfunction [8, 12]. H_2_S signals through posttranslational modifications of proteins known as *S*-sulfhydration and plays a role in metabolic and redox regulation [13–15].

Selenium shares very similar features as sulfur and has been strongly characterized as a micronutrient that walks a fine line between beneficial and toxic dosages [16, 17]. Keshan disease is a cardiomyopathy pathologically similar to chronic cardiac hypertrophy resulting in myocardial infarcture, which is actually attributed to the deficiency of selenium [18]. Although selenium-related cardiomyopathies are not entirely understood, it is believed that reduced antioxidant capabilities are strongly correlated [19, 20]. Selenium incorporation into selenoproteins is the cornerstone of antioxidant defence systems and therefore likely leads to altered redox signaling [21]. In the 3′-untranslated region of selenoprotein mRNA, there is a selenocysteine (Sec) insertion sequence (SECIS) that folds into a specific secondary structure, allowing for the recruitment of eukaryotic elongation factor selenocysteine (eEFSec) for facilitating Sec insertion in the stop codon of UGA [22]. To date, around 26 selenoproteins have been identified in mammals with just under half dedicated to antioxidant effects and redox signaling, including glutathione peroxidase (GPx), thioredoxin reductases (TrxR), and selenoprotein P (SePP1) [19, 23].

Hydrogen selenide (H_2_Se), the selenium homologue to H_2_S, is produced by the enzyme selenocysteine lyase (SCLY) for catalyzing the cleavage of Sec into H_2_Se and L-alanine [24, 25]. However, despite the similarities in structure and metabolism, selenium and sulfur systems share different chemical properties. Cys's pKa lies around 8.3 whereas Sec's is 5.2 and more than doubles Cys in its redox potential being -488 mV [24]. This high redox potential likely contributes to the effective involvement of selenoproteins in antioxidant defence, contributing to its hallmark reputation. Based on the homology and uniqueness of H_2_S and H_2_Se systems, both in their production and the similarities between their base element, it is reasonable to propose that H_2_Se may share some biological characteristics and functions of H_2_S, as a fourth gasotransmitter. In this study, we tested the interaction of H_2_S and H_2_Se systems in protection against cardiac hypertrophy as well as the underlying mechanisms.

## 2. Materials and Methods

### 2.1. Cell Culture

Rat cardiomyocytes (H9C2, American Type Culture Collection, Manassas, VA) were cultured in Dulbecco's modified Eagle medium supplemented with 10% heat-inactivated fetal bovine serum, 100 U/ml penicillin, and 100 mg/ml streptomycin at 37°C in a humidified atmosphere of 5% CO_2_. Every second day, the cells were washed with 1 ml Dulbecco's Phosphate-Buffered Saline (PBS) and the fresh media were added. H9C2 cells were cultured to a maximum of 80% confluence to avoid cellular differentiation.

### 2.2. Cell Viability Assay

The cell viability was measured based on the 3-(4,5-dimethylthiazol-2-yl)-2,5-diphenyltetrazolium bromide (MTT) assay as described previously [26]. H9C2 cells were seeded in a 96-well plate at a density of 15000 cells/well and allowed to sit overnight. After various treatments for 24 hours, MTT (0.5 mg/ml) in serum-free medium was added to each well and the plates were further incubated at 37°C for additional 4 hours. The MTT formazan was finally dissolved in 100 *μ*l dimethyl sulfoxide, following the absorbance measurement at 570 nm by a FLUOstar OPTIMA microplate spectrophotometer (BMG Labtech, Germany). The control cells with no treatment were considered as 100% viable, and the reading values were converted to the percentage of the control.

### 2.3. Measurement of the ROS Level

Fluorescent probe 2′,7′-dichlorodihydrofluorescein diacetate (DCFDA-H_2_) (Thermo Fisher Scientific, Ottawa, ON) was used to detect ROS [27, 28]. H9C2 cells were seeded in a 6-well plate and grown to 80% confluence. The cells were then incubated with or without 30 *μ*M NaHS or 0.3 *μ*M Na_2_Se for 30 minutes, washed with PBS, and incubated with 200 *μ*M H_2_O_2_ for 24 hours. After 24 hours, washing was avoided and the cells were treated with 10 *μ*M DCFDA-H_2_ (Invitrogen, Carlsbad, CA) in PBS for 15 minutes at 37°C. The plate was cooled to room temperature, and the fluorescence intensity was then measured by a FLUOstar OPTIMA at an excitation/emission of 485/515 nm, respectively, and images were also taken under an Olympus CX71 fluorescent microscope (Olympus, Tokyo, Japan). The cells were then collected to allow protein normalization. The fluorescence intensity was normalized by protein concentration and was expressed as the relative intensity compared to the control.

### 2.4. Cell Size Analysis

The cell surface area was determined by staining the cells with fluorophore-conjugated wheat germ agglutinin (WGA) [4, 29]. Briefly, H9C2 cells were seeded at a density of 10000 cells/plate in a 2 cm petri dish and left for 24 hours. The cells were incubated with or without 30 *μ*M NaHS or 0.3 *μ*M Na_2_Se for 30 minutes, washed with 1 ml PBS, and incubated with 200 *μ*M H_2_O_2_ for 24 hours. After that, the cells were washed with 1 ml PBS and fixed with 4% formaldehyde solution for 15 minutes, followed by two rounds of washing with 1 ml PBS. The cells were then stained with 1 ng/ml WGA coupled with a fluorophore in PBS for 45 minutes in the dark at room temperature. The cells were washed 2 times with 1 ml PBS followed by staining with DAPI, before being visualized under an Olympus CX71 fluorescent microscope (Olympus, Tokyo, Japan). Seven images were taken from each plate of cells from different areas to avoid bias. A minimum of 50 cells were surveyed using ImageJ software to determine the cell surface area and were normalized by cell number. The cells incubated with 100 *μ*M (±)-isoproterenol (ISO) for 24 hours acted as a positive control.

### 2.5. Measurement of Medium Se Level

H9C2 cells were seeded in a 6-well plate and grown to 80% confluence. The cells were then incubated with or without 30 *μ*M NaHS for 24 hours. After 24 hours, the cells and media were collected for protein normalization and Se analysis using 2,3-diaminonapthalene (DAN), respectively [30, 31]. A 300 *μ*l aliquot of the media was sampled and oxidized with an equal amount of 0.2% HNO_3_ (Thermo Fisher Scientific) for 15 minutes at 37°C. A 300 *μ*l of 15 mM DAN prepared in 0.1 N HCl was then added to the mixture and incubated for 15 minutes at 37°C on a shaker to obtain Se-DAN complex. The Se-DAN complex was extracted with 500 *μ*l cyclohexane, and the fluorescence intensity was measured in a clear F-bottom black 96-well plate using a FLUOstar OPTIMA at an excitation and emission of 385 and 515 nm, respectively. Fluorescence intensity was normalized by the amount of protein present per well and expressed relative to the control.

### 2.6. Measurement of GPx and TrxR Activities

To measure GPx activity, the cell lysates were incubated in 0.5 ml of a mixture containing 50 mm potassium phosphate buffer (pH 7.8), 1 mm EDTA, 1 mm NaN_3_, 10 mm GSH, and 2.4 units/ml glutathione reductase (GR) for 15 minutes. After addition of 10 *μ*l of 5 mm NADPH for 5 minutes followed by the addition of 10 *μ*l of 15 mm H_2_O_2_ for another 5 minutes, NADPH oxidation was then measured at 340 nm. The measured decrease in optical density at 340 nm was directly proportional to the enzyme activity in the sample. The assessment of TrxR activity was based on the enzymatic activity of TrxR to catalyze the reduction of 5,5′-dithiobis (2-nitrobenzoic) acid with NADPH to 5-thio-2-nitrobenzoic acid, which generates a strong yellow color with maximum absorbance at 412 nm. The activities of GPx and TrxR in the control sample were considered as 100%.

### 2.7. Western Blotting

After different treatments, cultured cells or mouse tissues were washed twice in ice-cold PBS and mixed in a lysis buffer (0.5 M EDTA, 1 M Tris-Cl at pH 7.4, and 0.3 M sucrose) in the presence of protease inhibitor cocktail (Sigma, St. Louis, MO) for sonication. An equal amount of proteins (50 *μ*g/well) was boiled in loading buffer for 5 minutes followed by separation by standard SDS/PAGE and then transferred onto polyvinylidene fluoride membranes (Pall Corporation, Pensacola, FL). Membranes were blocked with Tris-buffered saline (TBS) containing 3% nonfat milk at room temperature for 2 hours, then incubated overnight at 4°C with primary antibody on a shaker. The dilutions of primary antibodies were used as follows: SCLY (Abnova, Taipei, 1 : 1000), SePP1 (Boster, Pleasanton, CA, 1 : 1000), GPx1 (Santa Cruz Biotechnology, Santa Cruz, CA, 1 : 200), TrxR2 (Santa Cruz Biotechnology, 1 : 200), and GAPDH (Santa Cruz Biotechnology, 1 : 200). The membrane was then washed three times with TBS-Tween 20 (TBST) buffer and incubated in TBST solution with horseradish peroxidase-conjugated secondary antibody (diluted 1 : 5000) for 1 hour at room temperature on a shaker. Finally, the membrane was washed with TBST solution for 3 times. The immunoreactions were visualized with ECL (GE Healthcare, Amersham, UK) and exposed to X-ray film (Kodak Scientific Imaging film, Kodak, Rochester, NY).

The heart tissues were collected from 12-week-old CSE knockout mice and age-matched wild-type mice. All animal experiments were conducted in compliance with the Guide for the Care and Use of Laboratory Animals published by the US National Institutes of Health (NIH Publication No. 85-23, revised 1996) and approved by the Animal Care Committee of Laurentian University, Canada.

### 2.8. Real-Time PCR

H9C2 cells were incubated with or without 30 *μ*M NaHS or 0.3 *μ*M Na_2_Se for 30 minutes, washed with 1 ml PBS, and incubated with 200 *μ*M H_2_O_2_ for 24 hours. Total RNA from cells was isolated using Tri Reagent (Invitrogen, Carlsbad, CA). Briefly, the cells were sonicated in Tri Reagent, and total RNA was isolated using 200 *μ*l chloroform pelleted with 500 *μ*l isopropyl alcohol. The pellets were then washed with 100% ethanol and resuspended in RNase-free ddH_2_O. First strand cDNA was prepared by reverse transcriptase using a Maxima H Minus First Strand cDNA synthesis kit according to the manufacturer's protocol (Thermo Fisher Scientific). The quantification of mRNA transcript levels was performed with an iCycler iQ^5^ apparatus (Bio-Rad, Mississauga, ON) using the iCycler optical system software (version 3.1) with SYBR Green. Relative mRNA quantification was determined using the arithmetic formula “2^-*ΔΔ*CT^” where *Δ*CT is the difference between the threshold cycle of a given target cDNA and an endogenous reference of GAPDH gene [32]. The sequences of primers were used as follows: ANF (5′-AGCGGGGGCGGCACTTA-3′ and 5′-GGGCTCCAATCCTGTCAATCCTAC-3′), BNP (5′-CCTAGCCAGTCTCCAGAACAATCC-3′ and 5′-CTAAAACAACCTCAGCCCGTCACA-3′), GPx1 (5′-GGTTTCCCGTGCAATCAGTTCG-3′ and 5′-GGCACACCGGGGACCAAATG-3′), TrxR2 (5′-TCCCCTCCCTCATCAGAAAACTCC-3′ and 5′-GGCCGCCCCTCAGCAACAT-3′), SePP1 (5′-GGTTTGCCCTACTCCTTCCTCACT-3′ and 5′-CACTTGCCCCCATGTCTCAGC-3′), eEFSec (5′-ATGGGCCGTATGCTGTTCTTC-3′ and 5′-CAGCCGGCATGTGTTGGTGTGA-3′), glutamate-cysteine ligase modifier subunit (GCLM, 5′-CGCCTGCGGAAAAAGTG-3′ and 5′-GAGGGGAAGCCATGATGACAGAGT-3′), NDQ1 (5′-TGATTGTATTGGCCCACGCAGAG-3′ and 5′-GGCACCCCAAACCAATACAATG-3′), HO-1 (5′-CCCCCGAGGTCAAGCACAG-3′ and 5′-CACGGTCGCCAACAGGAAACT-3′), and GAPDH (5′-CACGGCAAGTTCAACGGCACAGT-3′ and 5′-AGCGGAAGGGGCGGAGATGAT-3′). All PCRs were performed in a volume of 20 *μ*l, including 2 *μ*l cDNA, 1 *μ*l each primer (1 *μ*M), 10 *μ*l SYBR Green PCR Master Mix, and 6 *μ*l nuclease-free water. The cycling was conducted at 95°C for 90 seconds followed by 38 cycles of 95°C for 10 seconds and at 60°C for 20 seconds. A standard melting curve analysis was performed at 95°C for 10 seconds followed at 55°C for 15 seconds and ramping to 95°C at 1° increments to confirm the absence of primer dimers.

### 2.9. Reagents

Unless otherwise stated, all reagents were purchased from Sigma (Oakville, ON) with the highest quality. All solutions were prepared in ddH_2_O, and all cellular incubation was performed in standard media unless stated.

### 2.10. Statistical Analysis

All data were presented as means ± SEM, representing at least 3 independent experiments. Statistical comparisons were made using Student's *t*-tests or one-way ANOVA followed by a post hoc analysis (Tukey test) where applicable. Values of *p* < 0.05 were considered to be statistically significant.

## 3. Results

### 3.1. H_2_S and H_2_Se Reverse H_2_O_2_-Induced Cell Death

**Figure 1:**
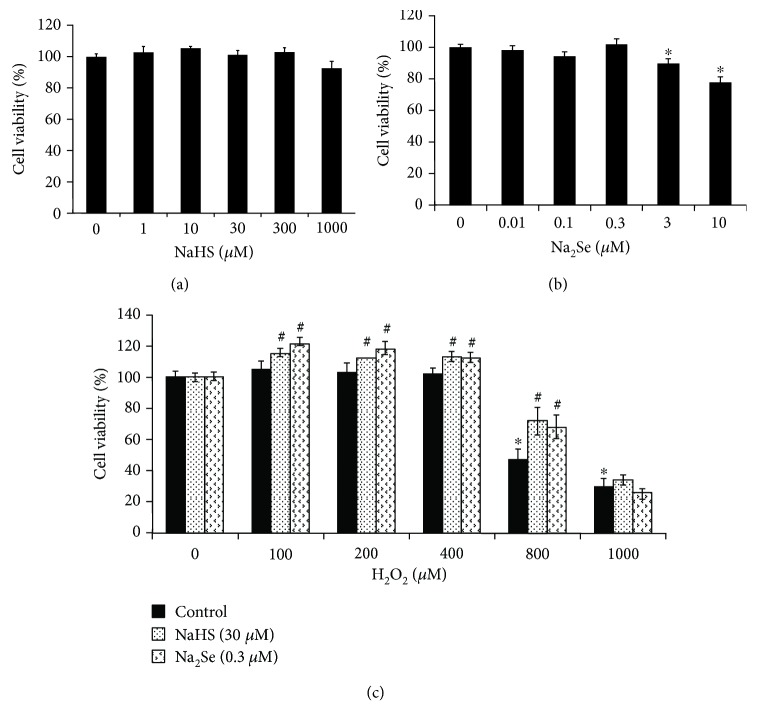
H_2_S or H_2_Se protects from H_2_O_2_-induced cell death. (a) Effect of H_2_S on cell viability. H9C2 cells were incubated with NaHS (0-1000 *μ*M) for 24 hours, and cell viability was measured with the MTT assay. (b) Effect of H_2_Se on cell viability. H9C2 cells were incubated with Na_2_Se (0-10 *μ*M) for 24 hours, and cell viability was measured with the MTT assay. ^∗^*p* < 0.05 versus control. (c) H_2_S or H_2_Se reverses H_2_O_2_-inhibited cell viability. H9C2 cells were treated with/without NaHS (30 *μ*M) or Na_2_Se (0.3 *μ*M) for 30 minutes prior to incubation with H_2_O_2_ (0-1000 *μ*M) for 24 hours. ^∗^*p* < 0.05 vs. control; ^#^*p* < 0.05 vs. H_2_O_2_ treatment alone in the same group. *n* = 4.

H9C2 cells treated with NaHS (1-1000 *μ*M) for 24 hours exhibited no change in cell viability (Figure 1(a)). A similar effect was viewed with cells treated by Na_2_Se (0-0.3 *μ*M) for 24 hours, while the signs of cellular toxicity started to appear at 3 *μ*M (Figure 1(b)). The cells treated with H_2_O_2_ exhibit significantly lower viability at 800 *μ*M and higher (Figure 1(c)). Pretreatment with NaHS (30 *μ*M) or Na_2_Se (0.3 *μ*M) for 30 minutes markedly reversed H_2_O_2_ (800 *μ*M)-induced cell death (Figure 1(c)), while NaHS or Na_2_Se did not reverse the higher dose of H_2_O_2_ (1000 *μ*M)-inhibited cell viability. Although H_2_O_2_ at a lower dose (100-400 *μ*M) did not cause cell death, coincubation of H_2_O_2_ with NaHS (30 *μ*M) or Na_2_Se (0.3 *μ*M) significantly stimulated cell growth.

### 3.2. H_2_S and H_2_Se Reverse H_2_O_2_-Induced Oxidative Stress and Cardiac Hypertrophy

**Figure 2:**
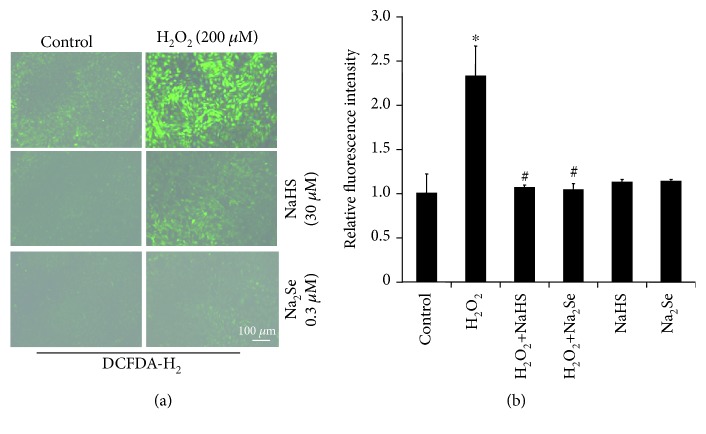
H_2_S or H_2_Se suppresses H_2_O_2_-induced oxidative stress. (a) Visualization of intracellular ROS using DCFDA-H_2_. (b) Intracellular ROS level analysis using DCFDA-H_2_ intensity relative to the control, normalized by the protein amount. H9C2 cells were pretreated with 30 *μ*M NaHS or 0.3 *μ*M Na_2_Se for 30 minutes, then incubated with 200 *μ*M H_2_O_2_ for additional 24 hours followed by staining with 10 *μ*M DCFDA-H_2_ for 15 minutes at 37°C. ^∗^*p* < 0.05 relative to the control; ^#^*p* < 0.05 compared to H_2_O_2_. *n* = 3.

**Figure 3:**
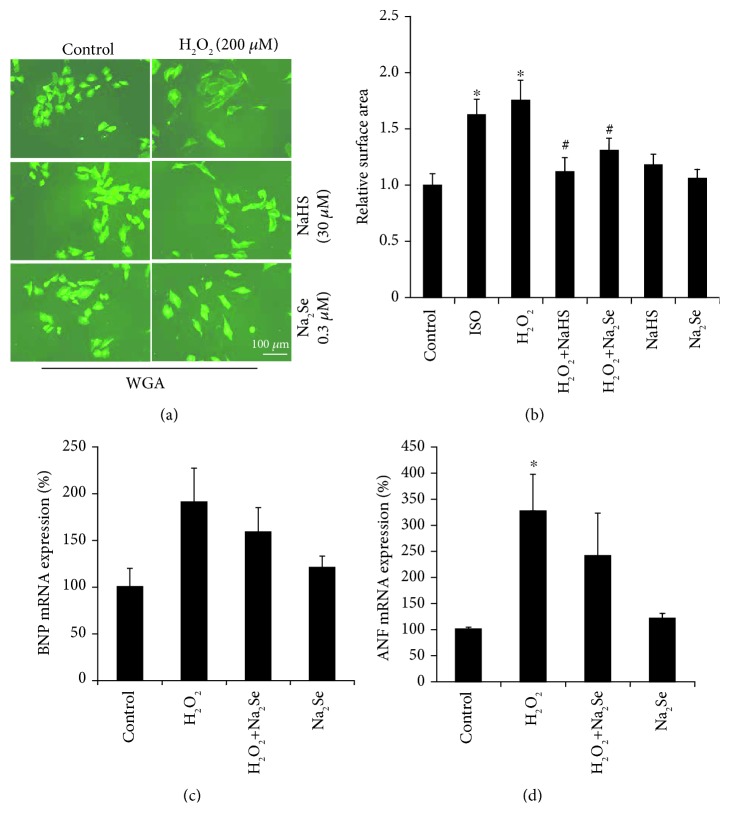
H_2_S or H_2_Se reverses H_2_O_2_-induced cell hypertrophy. H9C2 cells were pretreated with 30 *μ*M NaHS or 0.3 *μ*M Na_2_Se for 30 minutes, then incubated with 200 *μ*M H_2_O_2_ for additional 24 hours followed by staining with 1 ng/ml WGA coupled with a fluorophore for 45 minutes. Visualization of cell size (a) was observed with a fluorescence microscope, and the cell surface area was measured with ImageJ software (b). ^∗^*p* < 0.05 vs. control; ^#^*p* < 0.05 vs. H_2_O_2_ treatment. *n* = 3. (c, d) Induced mRNA expressions of ANF and BNP by H_2_O_2_ treatment. mRNA expression was analyzed by real-time PCR. ^∗^*p* < 0.05 vs. control. *n* = 3.

H9C2 cells treated with 200 *μ*M H_2_O_2_ showed increased signs of oxidative stress after 24 hours (Figure 2(a)), and internal ROS levels were increased 2.4-fold (Figure 2(b)). Pretreatment with H_2_S or H_2_Se significantly abolished the stimulatory role of H_2_O_2_-induced oxidative stress, while H_2_S or H_2_Se alone had no effect on the ROS level. We further observed that the cells treated with H_2_O_2_ (200 *μ*M) for 24 hours had a 2-fold increase in cell size when compared to control cells (Figures 3(a) and 3(b)). Pretreatment with either H_2_S or H_2_Se normalized cell size where H_2_S/H_2_Se itself had no effect. An increase in hypertrophy marker genes BNP (Figure 3(c)) and ANF (Figure 3(d)) was also observed in the cells treated with H_2_O_2_, which were partially reversed by coincubation with H_2_Se. ISO, a well-known inducer for heart cell hypertrophy, acted as a positive control here and also increased the cell surface area.

### 3.3. H_2_S Induces SLCY/H_2_Se Signaling

**Figure 4:**
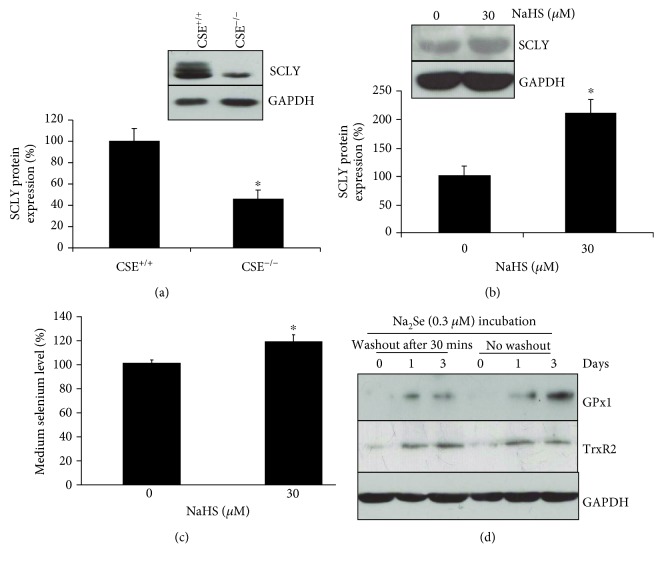
H_2_S stimulates SCLY/H_2_Se signaling. (a) CSE deficiency reduced SCLY protein expression in mouse heart tissues. The heart tissues were isolated from 12-week-old CSE knockout mice and wild-type littermates for analysis of SCLY protein expression by western blotting. *n* = 3. (b) H_2_S induces SCLY protein expression. H9C2 cells were treated with 30 *μ*M NaHS for 24 hours followed by western blotting detection of SCLY protein expression. *n* = 3. ^∗^*p* < 0.05. (c) H_2_S increases the Se level in media. After the cells were treated with 30 *μ*M NaHS for 24 hours, media were collected for analysis of the Se level by using DAN. *n* = 3. ^∗^*p* < 0.05. (d) H_2_Se induces the protein expressions of GPx1 and TrxR2. After the cells were incubated with 0.3 *μ*M Na_2_Se for 30 minutes, the cells were processed with or without selenide washout and continued to culture for 1-3 days; the protein expressions were detected with western blotting. *n* = 3.

To explore the interaction of H_2_S and H_2_Se, we first investigated the protein expression of SLCY in heart tissues from 12-week-old CSE knockout mice in comparison with age-matched wild-type mice. Lack of CSE expression and significantly lower production of endogenous H_2_S have been observed in the hearts of CSE knockout mice [8, 33]. The protein expression of SCLY was much lower in the heart tissue from CSE knockout mice, indicating the potential of H_2_S in regulating the contents of H_2_Se and intracellular Sec (Figure 4(a)). We then incubated H9C2 cells with 30 *μ*M NaHS for 24 hours to detect the change of SCLY protein expression. It was found that H_2_S also stimulated SCLY protein (Figure 4(b)). Moreover, extracellular selenium levels were also significantly higher after the cells were treated with H_2_S for 24 hours (Figure 4(c)). To investigate the direct effect of selenide on selenoprotein synthesis, we incubated cells with or without selenide washout for 1-3 days. As shown in Figure 4(d), Na_2_Se supplement induced the protein expressions of GPx1 and TrxR1 at day 1, which were not affected by either washout of selenide after 30 mins or continuous exposure to selenide. At day 3, GPx1 expression was further increased by continuous exposure of selenide but had a slight decrease after selenide washout after 30 mins. On the contrary, at day 3, the expression of TrxR1 had a slow drop by continuous exposure of selenide but kept higher after selenide washout. These data suggest that selenoprotein synthesis can be stimulated by the presence of either short-term (30 mins) or long-term (up to 3 days) incubation with selenide.

### 3.4. H_2_S Stimulates the Expressions and Activities of Selenoproteins

**Figure 5:**
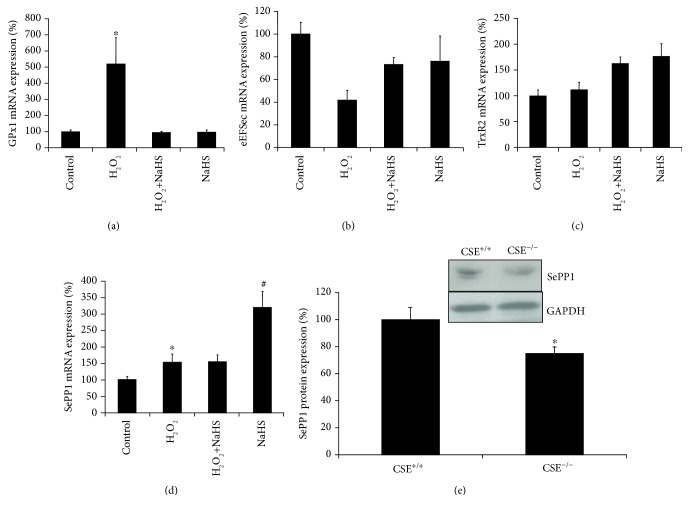
H_2_S regulates selenoproteins. H9C2 cells were pretreated with 30 *μ*M NaHS or 0.3 *μ*M Na_2_Se for 30 minutes, then incubated with 200 *μ*M H_2_O_2_ for additional 24 hours followed by real-time PCR analysis of GPx1 mRNA ((a) ^∗^*p* < 0.05 versus all other groups), TrxR2 mRNA (b), SePP1 mRNA ((c) ^∗^*p* < 0.05 versus control; ^#^*p* < 0.05 versus all other groups), and eEFSec mRNA ((e) ^∗^*p* < 0.05 versus control; ^#^*p* < 0.05 versus H_2_O_2_). *n* = 3. (d) CSE deficiency reduced SePP1 protein expression in mouse heart tissues. The heart tissues were isolated from 12-week-old CSE knockout mice and wild-type littermates for analysis of SCLY protein expression by western blotting. ^∗^*p* < 0.05.

**Figure 6:**
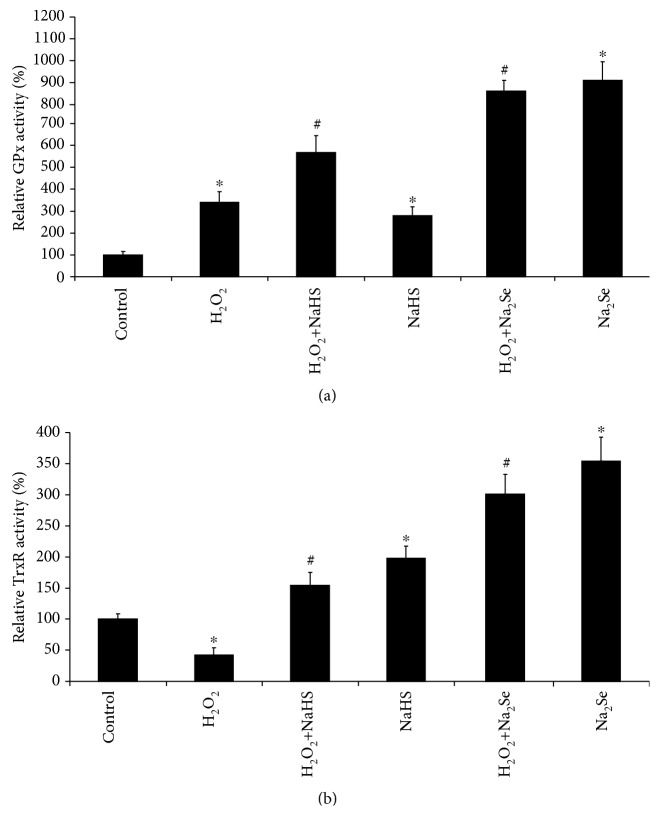
H_2_S/H_2_Se induces the activities of GPx and TrxR. H9C2 cells were pretreated with 30 *μ*M NaHS or 0.3 *μ*M Na_2_Se for 30 minutes, then incubated with 200 *μ*M H_2_O_2_ for additional 24 hours followed by measurement of GPx (a) and TrxR (b) activities. ^∗^*p* < 0.05 versus control; ^#^*p* < 0.05 versus H_2_O_2_. *n* = 4. The activity in control cells was considered as 100%.

**Figure 7:**
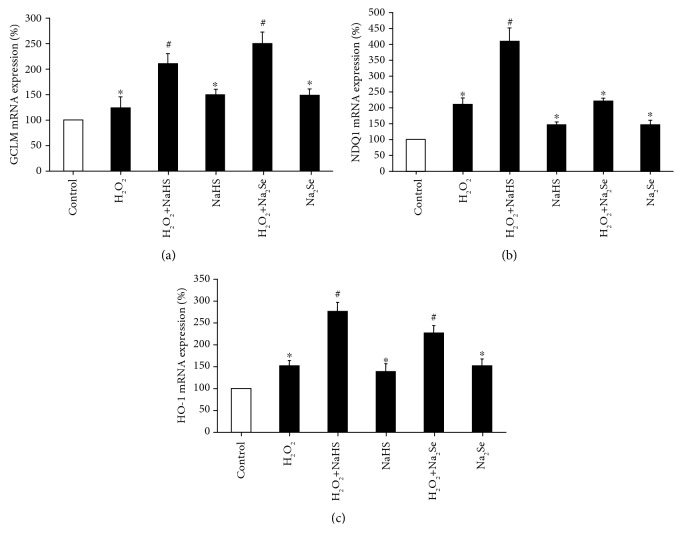
H_2_S/H_2_Se induces the mRNA expressions of Nrf2-target genes. H9C2 cells were pretreated with 30 *μ*M NaHS or 0.3 *μ*M Na_2_Se for 30 minutes, then incubated with 200 *μ*M H_2_O_2_ for additional 24 hours followed by real-time PCR detection of GCLM (a), NDQ1 (b), and HO-1 (c) mRNA expression. ^∗^*p* < 0.05 versus control; ^#^*p* < 0.05 versus H_2_O_2_. *n* = 3.

H9C2 cells treated with H_2_O_2_ for 24 hours had a significant 5-fold increase in GPx1 expression, which was normalized by H_2_S pretreatment (Figure 5(a)). eEFSec expression was reduced 2-fold by H_2_O_2_ treatment and restored with H_2_S pretreatment, while H_2_S alone had no effect (Figure 5(b)). TrxR2 was unchanged by H_2_O_2_ treatment; however, H_2_S increased TrxR2 expression both in the presence and absence of H_2_O_2_ (Figure 5(c)). SePP1 expression was significantly increased 1.5-fold by H_2_O_2_ and further increased 3-fold by H_2_S alone. H_2_S and H_2_O_2_ resulted in the same increase of SePP1 expression as did by H_2_O_2_ (Figure 5(d)). We also observed that the protein expression of SePP1 was significantly lower in the heart tissues from CSE knockout mice when compared with that from wild-type littermates (Figure 5(e)). In addition, NaHS/Na_2_Se enhanced the activities of both GPx and TrxR no matter the presence or absence of H_2_O_2_ (Figures 6(a) and 6(b)). Nrf2 is a master transcription factor driving the transcription of a large amount of antioxidant genes. We further validated that H_2_S or H_2_Se induced the mRNA expressions of classical Nrf2-target genes, including GLCM, NDQ1, and HO-1 (Figures 7(a)-7(c)), which provide additional protection against oxidative stress-caused cell hypertrophy.

## 4. Discussion

Lower doses of ROS usually contribute to chronic stress eventually leading to cellular apoptosis through metabolic starvation, while higher doses of ROS can lead to significant cell death within a short period of time [1]. A major finding of this study is that pretreatment with 30 *μ*M H_2_S or 0.3 *μ*M H_2_Se protects H9C2 cardiac cells from higher doses of H_2_O_2_ (800 *μ*M)-induced cellular death. We also observed that H_2_S or H_2_Se protects lower doses of H_2_O_2_ (200 *μ*M)-induced oxidative stress and cell hypertrophy by regulation of selenoproteins, a group of antioxidant proteins.

There is increasing evidence of H_2_S's ability to prevent cardiac hypertrophy at the physiologically relevant concentration [4, 5, 29, 34]. The potential of selenium in proper cardiac protection is not fully clear [35]. We report here for the first time the ability of pretreatment with H_2_Se to provide a protective effect against H_2_O_2_-induced cardiac cell hypertrophy. H_2_Se shared near identical results in terms of cell viability (Figure 1), ROS levels (Figure 2), and similar effects on ANF/BNP expression (Figure 3) as those previously reported for H_2_S [5, 34]. This provides strong evidence that H_2_Se is likely a downstream effect of H_2_S, or that H_2_Se may act in a similar fashion as a gasotransmitter, and that H_2_S and H_2_Se likely share some interactions and regulatory elements.

From the similarities between H_2_S and H_2_Se systems discussed above, we hypothesized that both systems would play a role in the regulation of one another. We demonstrated that heart tissue from CSE knockout mice had decreased protein expression of SCLY and SePP1 (Figures 4(a) and 5(e)), two enzymes crucial for intracellular H_2_Se production [36, 37]. A similar relationship was observed in H9C2 cells that treatment with H_2_S induced protein expression of SCLY and increased mRNA expression of SePP1. This provides evidence of the direct role of H_2_S in stimulating H_2_Se production within cardiac cells, which acts as the central metabolite for all selenoprotein synthesis. Treatment with H_2_S increased the concentration of selenium in the cultured medium as well as the activities of two selenoproteins, GPx and TrxR (Figure 4(c) and Figure 6), which indicates an increase in the bioavailability of H_2_Se. Similar to H_2_S, H_2_Se can diffuse freely through the cell membrane as it is a small uncharged molecule, follows concentration gradients, and is therefore likely responsible for this increase in its extracellular concentration. The increase in bioavailable H_2_Se can be used for further selenocysteine synthesis and thus selenoprotein translation such as SePP1 to increase selenocysteine distribution and storage [38]. Due to the higher lipid solubility of H_2_Se, short-term (30 minutes) incubation of the cells with selenide is able to induce the protein expression of selenoproteins over 3 days (Figure 4(d)).

Although it seems redundant to increase SCLY and SePP1 expression as the former degrades the latter, this can be explained through the localization of SePP1 in tissue [37]. H_2_S-producing genes and SePP1 are highly expressed in the heart and liver tissue as these organs appear to be the primary mode of selenium metabolism and storage [25, 39]. Higher levels of H_2_S increase SePP1 expression leading to a better distribution of SePP1 across an organism and yielding higher selenium content in plasma. As SePP1 levels increase in the plasma, it is likely that SePP1 will be selectively transported into tissues which require high levels of selenium for oxidative stress defence, such as neuronal tissues [23, 36, 38]. SePP1 has been shown to be selectively uptaken into neuronal tissue through apolipoprotein receptor following degradation by SCLY [23, 36]. This mode of transportation has been shown to be critical as the absence of SePP1 has been linked to severe neurological disorders, likely due to the diminished levels of selenium in the brain [23, 36]. The observed increased SePP1 expression caused by higher levels of H_2_S may be part of the whole organism's protective effect. Tissues high in selenium levels and SePP1 expression may be responsible for increasing the transportation of selenium to SePP1-dependent tissues, to “prime” their antioxidant defence systems upon detecting stressful conditions. Therefore, this increase in SePP1 may not be plausible at the cellular level but only at the tissue level which requires more research and investigation to confirm.

We also demonstrated that treatment with H_2_S and H_2_Se can reverse H_2_O_2_-induced oxidative stress, possibly through the regulation of selenoproteins, such as GPx1 and TrxR2. GPx1 expression is induced in response to high ROS levels causing changes in redox signaling, to attenuate ROS levels [40]. Here, we showed that H_2_O_2_ activates GPx1 mRNA expression, which is normalized by pretreatment with H_2_S (Figure 5(a)), indicating the increase in GPx1 as an adaptive response. TrxR2 mRNA expression was not affected by H_2_O_2_ but slightly increased by H_2_S. TrxR2 is an essential component of redox signaling, and therefore, its increase in mRNA expression with H_2_S likely acts as an upstream regulator of the observed protective effect. Previous studies have shown that H_2_S can also regulate cell processes that can effect redox signaling, in addition to directly regulating redox proteins themselves [34]. One possible mechanism of H_2_S regulating TrxR2 is through thyroid metabolism. Thyroid metabolism plays a key role in regulating cardiac health through thyroid hormone deiodinases which are also Sec containing proteins themselves [16, 41]. It is possible that H_2_S plays a role in regulating deiodinases through transcription or posttranslational modification followed by increased TrxR2 expression. A second mechanism of TrxR2 regulation could be contributed to H_2_Se through H_2_S activation. Stimulation of H_2_Se may enhance TrxR2 transcription through higher bioavailability of Se. H_2_Se may also function as a gasotransmitter like H_2_S, acting through a posttranslational modification of proteins [13–15]. H_2_Se is known to be heavily involved in the mitochondria in relation to ROS; therefore, it is possible that H_2_Se has an observable effect on TrxR2 regulation, through posttranslational modification, transcription activation, or redox signaling [42]. There are at least two types of TrxR, including TrxR1 and TrxR2. TrxR2, a mitochondrial thioredoxin reductase, plays a pivotal role in heart development. Heart-specific inactivation of TrxR2 results in fatal dilated cardiomyopathy, a condition reminiscent of that in Keshan disease [43]. However, the mice with a heart-restricted inactivation of TrxR1, the dominant cytosolic enzyme, develop normally and appear healthy [44]. These evidences strongly suggest the importance of TrxR2 but not TrxR1 in heart functions. Regardless the method of regulation, we provide clear evidence that H_2_S plays a regulatory role in TrxR2 and GPx1 expression/activity followed by a reduced ROS level and cell hypertrophy. Perhaps, even some functions of H_2_Se have been currently contributed to H_2_S. Further studies regarding the mechanistic regulation of TrxR2 and GPx1 via H_2_S and potentially H_2_Se must be considered.

Selenoprotein translation requires the SECIS region in the 3′UTR of the mRNA. Many studies have shown that the SECIS differs by selenoprotein, creating a hierarchy of expression and regulatory function [45]. eEFSec is a key factor for selenoprotein synthesis. While this study showed no change in eEFSec mRNA expression by H_2_S, excluding the possibility of H_2_S regulation of selenoprotein translation, it is also possible that H_2_S may posttranslationally modify eEFSec by *S*-sulfhydration, which would enhance eEFSec activity leading to higher selenoprotein translation [39]. This hypothesis needs to be tested in the future study. Besides enhanced selenoprotein synthesis, H_2_S/H_2_Se is also found to strengthen the transcriptions of a group of Nrf2-target antioxidant genes, including GCLM, NDQ1, and HO-1, suggesting that H_2_S or H_2_Se can protect the cardiomyocytes from oxidative stress-induced damage through multiple pathways [14, 46, 47].

**Figure 8:**
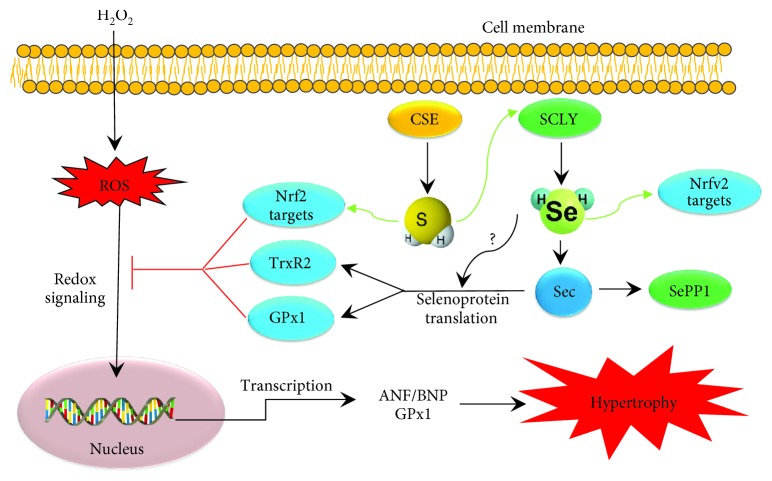
The proposed mechanism underlying the protective effect of H_2_S/H_2_Se against cardiac hypertrophy. H_2_S would enhance SCLY/H_2_Se signaling and regulate selenoproteins, a group of antioxidant proteins, which then lead to reduced oxidative stress and cell hypertrophy.

In conclusion, exogenous H_2_S stimulates SCLY protein expression and induces an increase of bioavailable Se content in H9C2 cells, and deficiency of CSE leads to a lower expression of SCLY and SePP1 expression in mouse heart tissue. Pretreatment with H_2_S or H_2_Se provides a protective effect against H_2_O_2_-induced oxidative stress, cell death, and cardiac hypertrophy. Mechanically, H_2_S would alter the expressions of selenoproteins by changing the SCLY/H_2_Se system and also enhance the transcriptions of Nrf2-targeted genes (Figure 8). Both H_2_S and H_2_Se signaling can be a target for therapeutic treatment of heart disorders.

## Data Availability

The data used to support the findings of this study are included within the article.

## Abbreviations

3MST: 3-Mercaptopyruvate sulfurtransferase
ANF: Atrial natriuretic factor
BNP: Brain natriuretic peptide
CBS: Cystathionine beta-synthase
CSE: Cystathionine gamma-lyase
CVD: Cardiovascular disease
Cys: L-cysteine
DAN: 2,3-Diaminonapthalene
eEFSec: Eukaryotic elongation factor selenocysteine
GLCM: Glutamate-cysteine ligase modifier subunit
GPx: Glutathione peroxidase
GR: Glutathione reductase
H_2_S: Hydrogen sulfide
H_2_Se: Hydrogen selenide
ISO: (±)-Isoproterenol
MTT: 3-(4,5-Dimethylthiazol-2-yl)-2,5-diphenyltetrazolium bromide
NaHS: Sodium hydrosulfide
ROS: Reactive oxygen species
SCLY: Selenocysteine lyase
Se: Selenium
Sec: Selenocysteine
SECIS: Selenocysteine insertion sequence
SePP1: Selenoprotein P
TrxR: Thioredoxin reductase
WGA: Wheat germ agglutinin
